# Synthesis, Characterization, X-ray Crystallography, Acetyl Cholinesterase Inhibition and Antioxidant Activities of Some Novel Ketone Derivatives of Gallic Hydrazide-Derived Schiff Bases

**DOI:** 10.3390/molecules17032408

**Published:** 2012-02-28

**Authors:** Nura Suleiman Gwaram, Hapipah Mohd Ali, Mahmood Ameen Abdulla, Michael J. C. Buckle, Sri Devi Sukumaran, Lip Yong Chung, Rozana Othman, Abeer A. Alhadi, Wageeh A. Yehye, A. Hamid A. Hadi, Pouya Hassandarvish, Hamid Khaledi, Siddig Ibrahim Abdelwahab

**Affiliations:** 1 Department of Chemistry, Faculty of Science, University of Malaya, Kuala Lumpur 50603, Malaysia; 2 Department of Molecular Medicine, Faculty of Medicine, University of Malaya, Kuala Lumpur 50603, Malaysia; 3 Departments of Pharmacy, Faculty of Medicine, University of Malaya, Kuala Lumpur 50603, Malaysia

**Keywords:** gallic hydrazide Schiff bases, AChE inhibition, antioxidant study, molecular docking

## Abstract

Alzheimer’s disease (AD) is the most common form of dementia among older people and the pathogenesis of this disease is associated with oxidative stress. Acetylcholinesterase inhibitors with antioxidant activities are considered potential treatments for AD. Some novel ketone derivatives of gallic hydrazide-derived Schiff bases were synthesized and examined for their antioxidant activities and *in vitro* and *in silico* acetyl cholinesterase inhibition. The compounds were characterized using spectroscopy and X-ray crystallography. The ferric reducing antioxidant power (FRAP) and 2,2-diphenyl-1-picrylhydrazyl (DPPH) assays revealed that all the compounds have strong antioxidant activities. *N*-(1-(5-bromo-2-hydroxyphenyl)-ethylidene)-3,4,5-trihydroxybenzohydrazide (**2**) was the most potent inhibitor of human acetyl cholinesterase, giving an inhibition rate of 77% at 100 μM. Molecular docking simulation of the ligand-enzyme complex suggested that the ligand may be positioned in the enzyme’s active-site gorge, interacting with residues in the peripheral anionic subsite (PAS) and acyl binding pocket (ABP). The current work warrants further preclinical studies to assess the potential for these novel compounds for the treatment of AD.

## 1. Introduction

The chemistry of hydrazones is an intensive area of study and numerous Schiff base ligands and their complexes of this type have been synthesized and their biological applications reported [1,2,3,4,5]. The major antioxidants currently used in foods are monohydroxy or polyhydroxy phenol compounds with various ring substitutions. These compounds have low activation energy for hydrogen donation [6]. Mainly from *in vitro* studies, polyphenols have been reported to have antioxidant [6,7,8], anti-cancer [9,10] and cardioprotecive activities [11].

Antioxidants such as vitamins A, E and C prevent the formation of free radicals and/or neutralize those that are formed, thus they break radical chains. They also repair the damage caused by free radicals, such as the DNA repair enzymes, e.g., transferases. Natural antioxidants are present in foods, but synthetic antioxidants may either be added to food to extend its shelf-life, or prepared by extraction from plant sources to be taken as supplements in concentrated form [8]. A number of studies have investigated a range of antioxidant agents in the hope of finding better and more effective treatments against AD [12]. Work has tended to focus on dietary antioxidants such as vitamins A, C, and E. Though these appear to have some benefits, results have proved frustratingly inconclusive [13]. Studies of many other dietary antioxidants polyphenols have also shown promise but, once more, their worth is yet unproven [14].

Researchers have recently investigated the potential health benefits of polyphenols in organic product [15]. Increased consumption of polyphenols has been associated with a reduced risk of cardiovascular disease and possibly cancer and stroke. Laboratory findings have shown that oxidative stress may play an important role to the pathogenesis of AD. Therefore, the risk of AD disease might be decreased by intake of antioxidants that neutralize the unfavorable effects of oxidative stress [16]. The present work reports the synthesis, characterization, antioxidants activities and X-ray crystal structures of Schiff bases derived from the condensation reaction of gallic hydrazide with pyridine and acetophenone derivatives, together with their acetylcholinesterase inhibition and antioxidant activity.

## 2. Results and Discussion

### 2.1. Chemistry

**Scheme 1:**
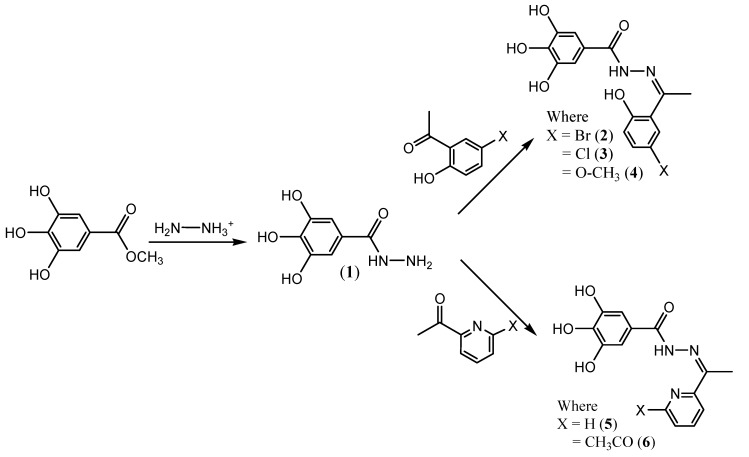
Reaction pathways.

The reaction of gallic hydrazide (**1**) with selected hydroxyacetophenones and pyridine derivatives resulted in the formation of the corresponding polyphenolic compounds: *N*-(1-(5-Bromo-2-hydroxyphenyl)-ethylidene)-3,4,5-trihydroxybenzohydrazide (**2**); *N*-(1-(5-Chloro-2-hydroxyphenyl)-ethylidene)-3,4,5-trihydroxybenzohydrazide (**3**); *N*-(1-(2-Hydroxy-5-methoxyphenyl)-ethylidene)-3,4,5-trihydroxybenzohydrazide (**4**); 3,4,5-Trihydroxybenzoic acid [1-pyridylethylidene] hydrazide (**5**); 3,4,5-Trihydroxybenzoic acid [1-(4-acetyl-pyridin-2-yl)-ethylidene] hydrazide (**6**) (Scheme 1). Their NMR, IR and UV-visible spectra were all consistent with the proposed structures.

The IR spectra of compounds **2–6** revealed the presence of both characteristic ketonic carbonyl absorptions (at wavenumbers of 1,618, 1,650, 1,674, 1,638 and 1,658 cm^−1^ respectively) and imine absorptions [17,18] (at wavenumbers of 1,621, 1,623, 1,610, 1,604, and 1,604 cm^−1^ respectively), thus confirming the formation of Schiff bases. The broad bands observed in the 3,570–3,390 cm^−1^ range were attributed to the phenolic hydroxyl groups [19]. Other series of bands in the wavenumber ranges 3,360–3,220 cm^−1^ and 1,110–950 cm^−1^ were assigned to υ(N-H) aliphatic and υ(N-N) stretching vibrations, respectively [20,21]. The ^1^H-NMR spectra of the Schiff bases revealed a singlet at δ 1.24, 1.24, 1.19, 2.12 and 1.24 ppm, respectively, which is due to a methyl group shielded by the imine moiety. Compound **4** also showed another singlet at δ 3.75 which was attributed to the methoxyl group. In the ^13^C-NMR spectra the signals in the range 167–152 ppm could be assigned to the azomethine carbon atoms [3].

### 2.2. X-ray Crystallography

**Figure 1:**
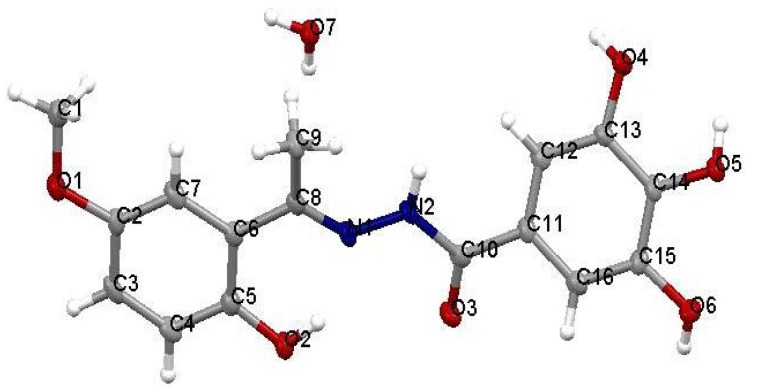
ORTEP-type view of the crystal structure of compound **4** showing the labeling scheme. Thermal ellipsoids are drawn at the 50% probability level.

The ORTEP view of the crystal structure of compound **4** is shown in Figure 1, and selected bonding parameters are listed in Table 1. The N1-N2 1.375(4), N1 = C8 1.294(4) and N2-C10 1.374(4) bond distances show no significant differences with those obtained previously for compound **2** [22]. For the description of the X-ray crystal geometry of the ligands, the molecule is proposed to be a planar or flat molecule. Planarity of the molecule makes it possible for the proton to be transferred through the hydrogen bond in the ground state with a small energy requirement.

**Table 1:** Selected bond distances (Å) and angles (°) for compound **4**.

Distances	
N1-N2	1.375(4)
N1-C8	1.294(4)
N2-C10	1.374(4)
C10-O3	1.231(4)
**Angles**	
C8-N1-N2	121.0(3)
C10-N2-N1	116.6(3)
O3-C10-N2	120.5(3)
O2-C5-C6	123.0(3)

The crystal data and structure refinement for compound **4** is summarized in Table 2.

**Table 2:** Crystal data and structure refinement for **4** and **5**.

Identification code	4	5
Empirical formula	C_16_H_18_N_2_O_7_	C_14_H_12_N_3_O_5_
Formula weight	350.32	302.27
Temperature/K	569(2)	296
Crystal system	Monoclinic	triclinic
Space group	P2_1_/n	P-1
a/Å	7.769(9)	6.6282(7)
b/Å	15.509(19)	9.9326(7)
c/Å	13.162(16)	12.8906(11)
α/°	90	79.982(2)
β/°	103.85(2)	80.995(2)
γ/°	90	76.5060(10)
Volume/Å^3^	1,540(3)	806.67(12)
Z	4	2
ρ_calc_mg/mm^3^	1.511	1.244
m/mm^‑1^	0.12	0.097
F(000)	736	314
Crystal size/mm^3^	0.28 × 0.05 × 0.01	0.33 × 0.14 × 0.11
2Θ range for data collection	4.14 to 50°	3.24 to 61.08°
Index ranges	−5 ≤ h ≤ 9, −18 ≤ k ≤ 18, −15 ≤ l ≤ 14	−8 ≤ h ≤ 6, −14 ≤ k ≤ 10, −17 ≤ l ≤ 17
Reflections collected	7,052	2,552
Independent reflections	2,704[R(int) = 0.0721]	2,237[R(int) = 0.0358]
Data/restraints/parameters	2,704/3/241	2,237/12/254
Goodness-of-fit on F^2^	0.986	0.804
Final R indexes [I >= 2σ (I)]	R_1_ = 0.0532, wR_2_ = 0.1121	R_1_ = 0.0462, wR_2_ = 0.1239
Final R indexes [all data]	R_1_ = 0.1117, wR_2_ = 0.1358	R_1_ = 0.0555, wR_2_ = 0.1354
Largest diff. peak/hole/e Å^−3^	0.305/−0.264	0.43/−0.34

### 2.3. Anti-AChE Assay

The inhibitory activities of compounds **1–6** on human acetyl cholinesterase were in the range of 16–77% at 100 μM (see Table 3) and thus comparable to those of the standard drugs tacrine and propidium.

**Table 3:** Human AChE inhibitory effects and anti-oxidant activities for compounds **1–6**.

Compounds	Molecular weight	AChE Inhibition (%)(Final conc. = 1 × 10^−4^ M)	DPPH(IC_50_, μg/mL)	FRAP value(Mean ± SD)
**1**	184.15	38.0 ± 1.3	1.210 ± 0.002	81,633.30 ± 0.075
**2**	381.18	77.0 ± 1.8	1.140 ± 0.001	62,200.00 ± 0.083
**3**	336.73	68.9 ± 1.8	1.400 ± 0.002	35,740.00 ± 0.011
**4**	332.31	48.5 ± 2.5	1.220 ± 0.001	30,080.00 ± 0.054
**5**	287.27	16.4 ± 1.4	1.460 ± 0.001	22,946.70 ± 0.004
**6**	329.31	71.5 ± 1.7	2.300 ± 0.001	23,340.00 ± 0.021
Propidium	-	54.5 ± 1.6	-	-
Tacrine	-	51.2 ± 1.6	-	-
Ascorbic acid	-	-	2.260 ± 0.001	19,400.00 ± 0.007
BHT	-	-	-	187.3 ± 2.6

**1**: Gallic hydrazide; **2**: *N*-(1-(5-Bromo-2-hydroxyphenyl)-ethylidene)-3,4,5-trihydroxybenzohydrazide; **3**: *N*-(1-(5-Chloro-2-hydroxyphenyl)-ethylidene)-3,4,5-trihydroxybenzohydrazide; **4**: *N*-(1-(2-Hydroxy-5-methoxyphenyl)-ethylidene)-3,4,5-trihydroxybenzohydrazide; **5**: 3,4,5-Trihydroxybenzoic acid [1-pyridylethylidene] hydrazide, **6**: 3,4,5-Trihydroxybenzoic acid [1-(4-acetyl-pyridin-2-yl)-ethylidene] hydrazide; BHT: Butylated hydroxytoluene.

Compounds **2**, **3** and **6** showed the highest activities. This indicates that introduction of chlorine or bromine atom at position 5 of the acetophenone moiety significantly enhances the inhibition activity. This might be ascribed to the electron donating properties of the halogens by resonance, making the lone pair electrons more available to a plausible electron transfer. The activity of **6** could be attributed to the presence of the acetyl group on the pyridine ring making the lone pair electron on the pyridine nitrogen atom available for electron transfer, also increasing of bond dissociation enthalpy (BDE) values in phenolic structure contain substituent of electron withdrawing groups such as COR, COOR, CN [23] could discourage the abstraction of hydrogen. O-H bond dissociation enthalpy (BDE) is a theoretical parameter successfully used to measure the H-atom-donating ability of various antioxidants. Similar results have been reported by Kadoma [24].

### 2.4. Molecular Docking

The crystal structure of hAChE (in complex with fasciculin-2) (pdb id: 1B41) shows that the enzyme possesses a deep narrow gorge which penetrates halfway into the enzyme, where the catalytic site resides [25]. The binding site of AChE consists of five subsites: a peripheral anionic site (PAS), an acyl binding pocket (ABP), the esteratic site (ES), an oxyanion hole (OH) and an anionic subsite (AS).

**Figure 2:**
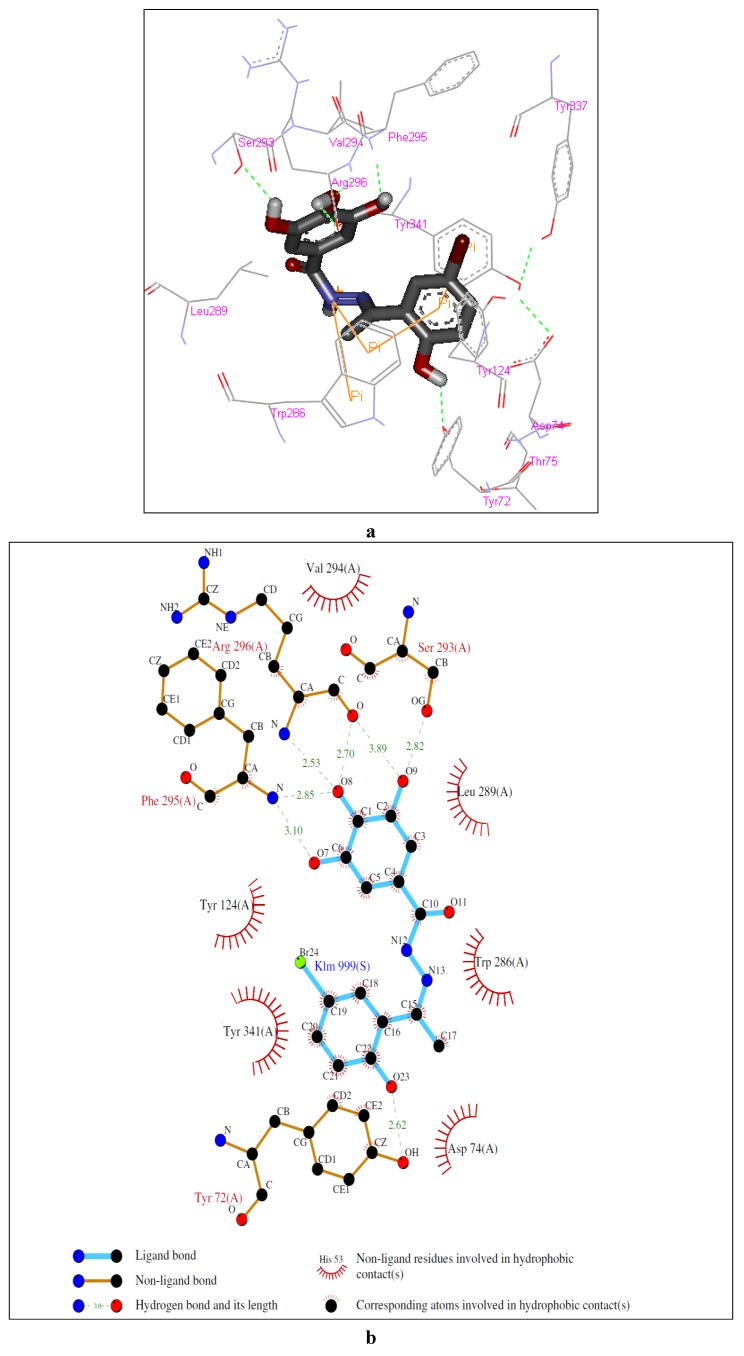
Representations of the molecular model of the complex formed between compound **2** and hAChE. (**a**) 3D representation of the ligand-enzyme binding interactions. Compound **2** is represented as a dark grey sticks and hydrogen bonds as green dashed lines; (**b**) 2D schematic representation of the hydrogen bonding and hydrophobic interactions.

The residues that have been reported to be involved in protein-ligand interactions are Tyr 72, Asp 74, Tyr 124, Ser 125, Trp 286, Tyr 337 and Tyr 341 (PAS); Trp 236, Phe 295, Phe 297 and Phe 338 (ABP); Ser 203, His 447 and Glu 334 (ES); Gly 121, Gly 122 and Ala 204 (OH); and Trp 86, Tyr 133, Glu 202, Glu 448 and Ile 451 (AS) [26]. The molecular docking simulation of the complex formed between compound **2** and hAChE (Figure 2) showed the ligand well positioned in the active-site gorge, with the monohydroxyphenyl and trihydroxyphenyl moieties interacting with residues in the PAS and ABP, respectively.

A closer inspection of the interactions at the PAS showed the presence of a hydrogen bond between the 2-hydroxyl group and Tyr 72, *π-π* stacking involving the monohydroxyphenyl ring, Trp 286 and Tyr 341 and a cation-*π* interaction between the protonated nitrogen atom of the amide and Trp 286. Furthermore, hydrophobic interactions between **2** and the rich aromatic residues (Asp 74, Tyr 124, Trp 286, Leu 289 and Tyr 341) along the gorge appear to direct the trihydroxyphenyl moiety into the ABP, thus enabling the phenolic hydroxyl groups to form a network of hydrogen bonds with Ser 293, Phe 295 and Arg 296.

**Figure 3:**
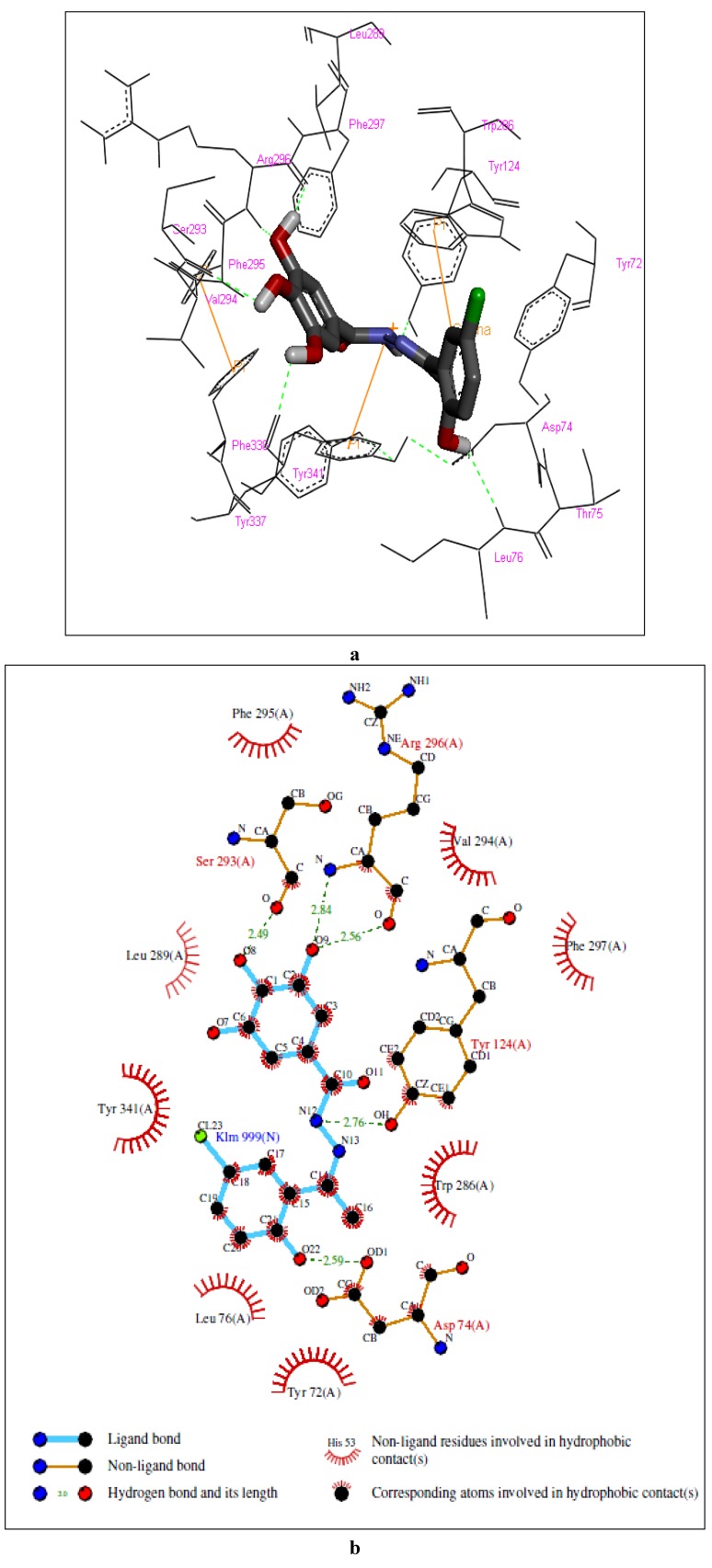
Representations of the molecular model of the complex formed between compound **3** and hAChE. (**a**) 3D representation of the ligand-enzyme binding interactions. Compound **3** is represented as a dark grey sticks and hydrogen bonds as green dashed lines; (**b**) 2D schematic representation of the hydrogen bonding and hydrophobic interactions.

**Figure 4:**
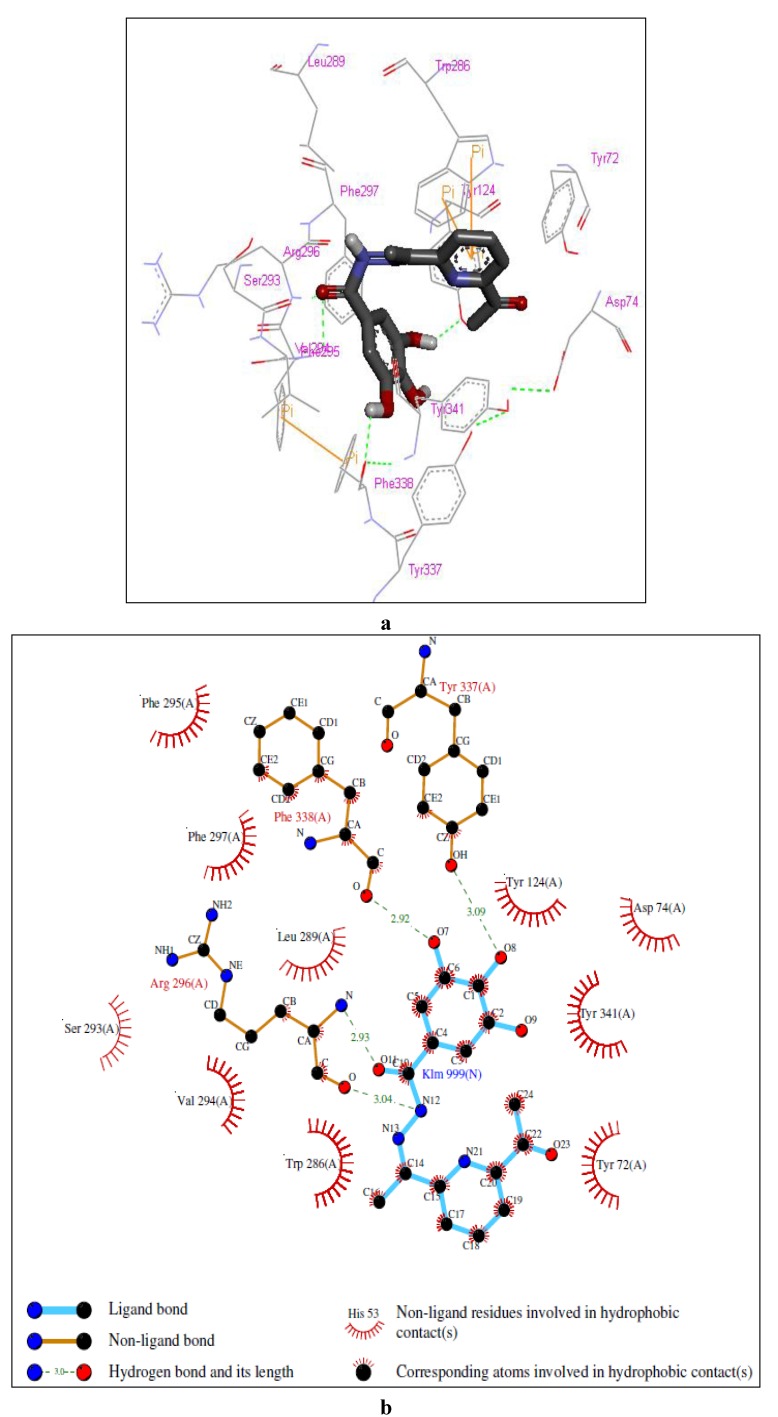
Representations of the molecular model of the complex formed between compound **6** and hAChE. (**a**) 3D representation of the ligand-enzyme binding interactions. Compound **6** is represented as a dark grey sticks and hydrogen bonds as green dashed lines; (**b**) 2D schematic representation of the hydrogen bonding and hydrophobic interactions.

Molecular modeling of the complexes formed between the enzyme and compounds **3** and **6** suggested the involvement of a similar set of interactions as for the complex with compound **2** (see Figure 3 and Figure 4). In the case of the complex with compound **3**, the model showed, at the PAS, a hydrogen bond between the 2-hydroxyl group and Asp 74, a *σ-π* interaction between carbon 6 in the aromatic ring and Trp 286, a cation-*π* interaction between the protonated nitrogen atom of the amide and Tyr 341 and a hydrogen bond between the amide nitrogen atom and Tyr 124 and, in the ABP, hydrogen bonds between two of the hydroxyl groups in the trihydroxyphenyl moiety and Ser 293 and Arg 296. The complex with compound **6** showed, at the PAS, *π-π* stacking between the pyridinyl ring and Trp 286 and hydrogen bonds between the amide nitrogen atom and the carbonyl group and Arg 296 and, in the ABP, hydrogen bonds between of the hydroxyl groups in the trihydroxyphenyl moiety and Tyr 337 and Phe 338.

This analysis suggests that the hAChE inhibition activity of compounds **2**, **3** and **6** is probably due to their ability to block the active-site gorge, thus preventing the substrate, acetylcholine, from entering the active site.

### 2.5. Antioxidant Assays

The antioxidant efficacies of the compounds **1–6** were tested and the results obtained (see Table 3) revealed differing activities in the two assays. This indicates that two mechanisms, operating in different ways, must be responsible for the observed activity. The color change from deep purple to yellow at 515 nm observed in the DPPH assay confirmed the radical scavenging activity of the compounds. A reference curve of absorbance (A) against DPPH concentration in methanol was plotted and used for the calculation of DPPH concentration at various reaction times (R^2^ = 0.9999). The compounds showed IC_50_ values in the range of 1.1–2.3 μg/mL. All the compounds tested showed a lower IC_50_ compared to the positive control used (ascorbic acid), except for compound **6**, which showed no significant difference with the IC_50_ of the positive control. The high activity of the compounds in the DPPH assay can be related to the resonance effect of the polyhydroxyl groups attached to the phenolic ring in the compounds, whereupon electron donating substituents increase the electron density in the aromatic ring making it more reactive towards electrophilic attack or mainly due to their redox properties, which can play an important role in the absorption and neutralization of free radicals, the quenching of singlet and triplet oxygen, or the decomposition of peroxides [27,28]. This presumably promotes the release of phenolic hydrogen to the (1,1-diphenyl-2-picrylhydrazyl) free radical indicated by a color change from purple to yellow. The second method used for testing the antioxidant activities of these compounds was the FRAP assay. It is considered an accurate method for assessing “antioxidant power”. Ferric to ferrous ion reduction at low pH causes a colored ferrous-tripyridyltriazine complex to form. FRAP values are obtained by comparing the absorbance change at 593 nm in test reaction mixtures with those containing ferrous ions at known concentrations.

In this study, the compounds showed FRAP values in the range 2,000–9,000 which is above the values shown by BHT and ascorbic acid used as standards. It was observed that compounds **1–5** demonstrated the highest activities in the DPPH assay while **1** and **2** showed the highest values in the FRAP assay. This can be attributed to increased π-electron delocalization within the pyridine ring which increases the electron density and causes ferric ion reduction [29].

## 3. Experimental Section

### 3.1. General

The compounds synthesized in this study were characterized by spectral methods. IR spectra was recorded at the wavelength range from 4,000–400 cm^−1^ using a Perkin Elmer 783 spectrophotometer, NMR spectra were obtained on a ECA400 FT-NMR spectrophotometer using TMS as internal standard, UV-visible spectra were recorded on a UV-1650PC model UV-visible spectrophotometer, Melting points were measured using a Gallenkamp melting point apparatus and are Elemental analysis was conducted on Costech Elemental Combustion System CNHS-O elemental analyzer. General grade solvents and reagents were used unless stated otherwise and were obtained from Aldrich Chemicals UK Ltd. and Acros Ltd. (UK). Methyl-3,4,5-trihydroxybenzoate, hydrazine hydrate, 2-hydroxy-5-methoxyacetophenone, 5-bromo-2-hydroxyacetophenone, 5-chloro-2-hydroxyacetophenone, 2-cetylpyridine, 2,6-diacetylpyridine, Dilute Hydrochloric acid (0.01 M), Distilled Ethanol. Distilled water and Dimethyl formamide (DMF).

### 3.2. Gallic Hydrazide *(**1**)*


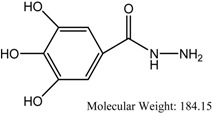


An ethanolic solution (20 mL) containing methyl 3,4,5-trihydroxybenzoate (1.84 g, 0.01 M C_8_H_8_O_5_) and hydrazine (9 mL) was stirred for 30 minutes, until all the solute completely dissolved then distilled ethanol (45 mL) was added. The mixture was refluxed for about 6–8 h. The resulting white precipitate was collected by filtration, washed several times with distilled water and then dried under vacuum. Yield = 70%, melting point = 290 °C, elemental analysis theory: C (45.6%); H (4.3%); N (15.2%); found: C (45.2%); H (5.04%); N (15.04%), %, FT-IR spectra (KBr); 3,429 cm^−1^ (νAr-OH), 3,299 cm^−1^ (νN-H), 1,654 cm^−1^ (νC=O), 1,344cm^−1^ (νC-O), 1,103 cm^−1^ (νN-N), ^1^H-NMR (DMSO-d_6_): 9.35 ppm [δ(Ar-OH), 1H s], 9.13 ppm, 9.05 ppm [δ(Ar-OH), 2H, d], 8.65 ppm [δ(NH), 1H, brd], 6.79 ppm, 6.82 ppm [δ(Ar-H), 2H s], 4.37 ppm [δ(NH_2_), 2H s]. ^13^C-NMR (DMSO-d_6_): 166.39 ppm [δ(CONH), 1C], 145.37 ppm [δ(aromatic), 1C-OH], 136.10, 136.42 ppm [δ(aromatic), 2C-OH], 124.00 ppm [δ(aromatic), 1C], 106.43 ppm [δ(aromatic), 2C = C] ppm.

### 3.3. *N*-(1-(5-Bromo-2-hydroxyphenyl)-ethylidene)-3,4,5-trihydroxybenzohydrazide *(**2**)*


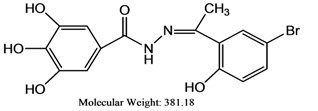


Gallic hydrazide (1.84 g, 0.01 M) in (20 mL) ethanol was added to an ethanolic solution (20 mL) of 5-bromo-2-acetophenone (2.15 g, 0.01 M respectively). The mixture was stirred for 2–3 h whereupon the color of the solution turned yellowish. The pH was adjusted by adding a few drops of dilute HCl. The reaction was continued for another hour resulting in the formation of a yellow precipitate. More precipitate was obtained when reducing the solvent by distillation. The product was collected by filtration, washed several times with ethanol and dried in an oven. (Yield 75%), elemental analysis: theory C (47.50); H (3.89); N (7.32); found C (47.26); H (3.44); N (7.35); IR spectra (KBr); 3,558 cm^−1^ (νAr-OH), 3,235 cm^−1^ (νN-H), 1,604 cm^−1^ (νC=N), 1,658 cm^−1^ (νC=O), 1,242 cm^−1^ (νC-O), 953 cm^−1^ (νN-N), ^1^H-NMR (DMSO-d_6_): 13.57 ppm [δ(OH), 1H, s], 11.10 ppm [δ(OH), 1H, s], 9.05 ppm [δ(OH), 2H, brd], 6.79–7.73 ppm [δ(aromatic), 5H, m], 4.34 ppm [δ(NH), 1H, s], 1.24 ppm [δ(-CH3 ), 3H, s]. ^13^C-NMR (DMSO-d_6_): 166.39 ppm [δ(C=N)], 164.43 ppm [δ(C=O)], 145.53 ppm, 145.36 ppm [δ(aromatic), 2C-OH], 137.41 ppm [δ(aromatic), 1C-OH], 137.40, 136.10 ppm, [δ(aromatic), 2C], 130.31 ppm [δ(aromatic), 1C], 123.51 ppm, 122.35 ppm, 121.52 ppm, 119.50 ppm [δ(aromatic), 4C], 107.54 ppm, 106.42 ppm [δ(aromatic), 2C=C], 13.92 ppm [δ(CH_3_)] ppm.

### 3.4. *N*-(1-(5-Chloro-2-hydroxyphenyl)-ethylidene)-3,4,5-trihydroxybenzohydrazide *(**3**)*


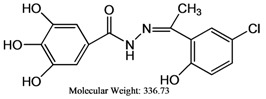


An accurately weighed amount of gallic hydrazide (1.84 g, 0.01 M) in ethanol (20 mL) was added to the same ethanolic solution (20 mL) of 5-chloro-2-hydroxyacetophenone (2.15 g, 0.01 M), and the mixture was stirred for 2–3 h as the color of the solution turned yellowish. The pH was adjusted by adding few drops of dilute HCl. The reaction was continued for another 1 h to give a yellow precipitate. More precipitate was obtained after reducing the solvent by distillation. The product was collected by filtration, washed several times with ethanol and dried in an oven. (Yield 75%) respectively, elemental analysis: theory C (53.50); H (3.89); N (8.32); found C (53.39); H (3.76); N (8.33); IR spectra (KBr); 3,568 cm^−1^ (νAr-OH), 3,225 cm^−1^ (νN-H), 1,638 cm^−1^ (νC=O), 1,604 cm^−1^ (νC=N), 1,212 cm^−1^ (νC-O), 953 cm^−1^ (νN-N). ^1^H-NMR (DMSO-d_6_): 11.14 ppm [δ(OH), 1H, s], 11.13 ppm [δ(OH), 1H, s], 9.30 ppm [δ(OH), 2H, brd], 7.66 ppm [δ(NH), 1H, s], 7.37–6.93 ppm [δ(aromatic), 3H, m], 6.983 ppm, 6.976 ppm [δ(aromatic), 2H str d], 2.12 ppm [δ(-CH3), 3H, s]. ^13^C-NMR (DMSO-d_6_): 166.94 ppm [δ(C=N)], 164.84 ppm [δ(C=O)], 157.94 ppm [δ(aromatic) 1C], 146.10 ppm, 145.93 ppm [δ(aromatic), 2C-OH], 137.97 ppm [δ (aromatic), 1C-OH], 128.10 ppm, 124.08 ppm, 122.92 ppm, 122.53 ppm, 121.48 ppm, 119.58 ppm [δ(aromatic), 6C], 108.10 ppm, 106.98 ppm [δ(aromatic), 2C=C], 14.48 ppm [δ(CH_3_)] ppm.

### 3.5. *N*-(1-(2-Hydroxy-5-methoxyphenyl)-ethylidene)-3,4,5-trihydroxybenzohydrazide *(**4**)*


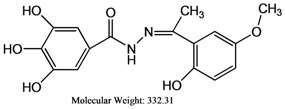


An ethanolic solution (20 mL) of gallic hydrazide (1.84 g, 0.01 M) was added to an ethanolic solution (20 mL) of 2-hydroxy-5-methoxyacetophenone (1.06 g, 0.01 M) in 1:1 ratio. The mixture was refluxed for 2–3 h resulting in the formation of a slightly yellow precipitate. More precipitate was obtained by removal of some solvent by distillation. The product was collected by filtration, washed several times with ethanol until a milky colored compound is obtained. The ligand was re-crystallized by using the same solvent (ethanol), filtered to remove the suspended impurities and a single crystal was obtained suitable for X-ray analysis. (Yield = 65%), elemental analysis: theory C (57.83); H (4.85); N (8.43); found C (58.30); H (4.51); N (8.71); IR spectra (KBr); 3,467 cm^−1^ (νAr-OH), 3,308 cm^−1^ (νN-H), 1,650 cm^−1^ (νC=O), 1,623 cm^−1^ (νC=N), 1,282 cm^−1^ (νC-O), 944 cm^−1^ (νN-N), ^1^H-NMR (DMSO-d_6_): 12.89 ppm [δ(OH)], 10.98 ppm [δ(OH), 1H, s], 9.28 ppm [δ(OH), 2H, brd], 8.90 ppm [δ(NH)], 7.11 ppm [δ(aromatic), 2H str s], 7.11–6.83 ppm [δ(aromatic), 3H, m], 3.75 ppm [δ(O-CH_3_), 3H, s], 1.24 ppm [δ(-CH3), 3H, s]. ^13^C-NMR (DMSO-d_6_): 152.58 ppm [δ(C=N)], 151.36 ppm [δ(C=O)], 145.52 ppm [δ(aromatic), 2C-OH], 137.27 ppm [δ(aromatic), 1C-OH], 122.70 ppm, 122.61 ppm, 117.77 ppm, 117.39 ppm, 119.49 ppm [δ(aromatic), 5C], 107.44 ppm [δ(aromatic), 2C=C], 55.54 ppm [δ(O-CH3)], 13.94 ppm [δ(CH_3_)] ppm.

### 3.6. 3,4,5-Trihydroxybenzoic Acid [1-Pyridylethylidene] Hydrazide *(**5**)*


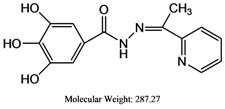


A stoichiometric amount of gallic hydrazide (1.84 g, 0.01 M) in ethanol (20 mL) was added to a solution (20 mL) of 2-acetylpyridine (1.21 mL, 0.01 mmol) and the mixture was refluxed on a water bath for 2–3 h, resulting in the formation of a small amount of white precipitate. More precipitate was obtained after evaporating the solvent by distillation. The product was then collected by filtration, washed several times with ethanol until a clear white powdery product was formed. The white powdered ligand was recrystallized by using DMF to obtain single crystals for X-ray structural determination. (Yield = 65%), melting point = 253 °C, elemental analysis: theory: C (58.53%); H (4.56%); N (14.63%); found: C (57.99%); H (4.56%); N (14.06%), IR spectra (KBr); 3,399 cm^−1^ (νAr-OH), 3,351 cm^−1^ (νN-H), 1,621 cm^−1^ (νC=N), 1,618 cm^−1^ (νC=O), 1,560 cm^−1^ (νC=N)Py, 1,281 cm^−1^ (νC-O), 1,032 cm^−1^ (νN-N), ^1^H-NMR (DMSO-d_6_): 9.38 ppm [δ(OH), 1H, s], 9.21 ppm, 9.11 ppm [δ(OH), 2H, brd], 8.14–8.07 ppm [δ(aromatic), 2H m], 7.87–7.70 ppm [δ(aromatic), 2H m], 7.63 ppm [δ(NH), 1H, s, brd], 6.92 ppm [δ(aromatic), 2H str s], 1.24 ppm [δ(-CH3), 3H, s]. ^13^C-NMR (DMSO-d_6_): 162.47 ppm [δ(C=N)], 155.25 ppm [δ(C=O)], 148.54 ppm [δ(aromatic), pyridine], 145.85 ppm [δ (aromatic), 2C-OH], 138.70 ppm [δ(aromatic), 1C-OH], 136.52 ppm [δ(aromatic), pyridine], 124.57 ppm [δ(aromatic), 1C], 123.95 ppm, 122.90 ppm [δ(aromatic), pyridine], 107.55 ppm, 106.35 ppm [δ (aromatic), 2C=C], 22.10 ppm [δ(CH_3_)] ppm.

### 3.7. *N'*-(1-(6-Acetylpyridin-2-yl)ethylidene)-3,4,5-trihydroxybenzohydrazide *(**6**)*


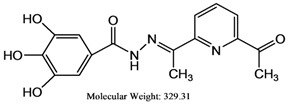


A weighed amount of gallic hydrazide (1.84 g, 0.01 M) in (20 mL) ethanol was added to the same volume (20 mL) of ethanolic solution of 2,6-diacetylpyridine (1.63 g, 0.01 M). The mixture was stirred for 2–3 h while the color of the solution turned yellowish. The pH was adjusted by adding few drops of dilute HCl. The reaction was continued for another one hour resulting in the formation of a yellow precipitate. The product was collected by filtration, washed several times with ethanol and dried in an oven. (Yield 90%), elemental analysis: theory C (58.36); H (4.59); N (12.76); found: C (57.99); H (4.89); N (13.11); IR spectra (KBr); 3,448 cm^−1^ (νAr-OH), 3,239 cm^−1^ (νN-H), 1,674 cm^−1^ (νC=O), 1,610 cm^−1^ (νC=N), 1,511 cm^−1^ (νC-N)py, 1,265 cm^−1^ (νC-O), 955cm^−1^ (νN-N). ^1^H-NMR (DMSO-d_6_): 9.29 ppm [δ(OH), 1H, s], 8.26 ppm, 8.28 ppm [δ(OH), 2H, brd], 8.14–8.03 ppm [δ(aromatic), 2H m], 8.01–7.90 ppm [δ(aromatic), 2H m] 6.89 ppm [δ(aromatic), 2H str s], 4.3 ppm [δ(NH), 1H, s], 1.19 ppm [δ(-CH3), 3H, s]. ^13^C-NMR (DMSO-d_6_): 166.38 ppm [δ(C=N)], 164.34 ppm [δ(C=O)], 151.97 ppm [δ(aromatic), pyridine], 145.37 ppm [δ(aromatic), 2C-OH], 137.87 ppm [δ(aromatic) 1C-OH], 136.13 ppm [δ(aromatic), pyridine], 123.87 ppm [δ(aromatic), 1C], 123.51 ppm [δ(aromatic), 2C=C], 121.13 ppm, 120.26 ppm [δ(aromatic), pyridine], 199.24 ppm, 106.45 ppm [δ(C=OCH3)], 25.50 ppm [δ(CH_3_-C=O)], 18.52 ppm [δ(CH_3_)] ppm.

### 3.8. X-ray Crystallography

Diffraction data were measured using a Bruker SMART Apex II CCD area-detector diffractometer (graphite-monochromated Mo K radiation, = 0.71073 Å). The orientation matrix, unit cell refinement and data reduction were all handled by the Apex2 software (SAINT integration, SADABS absorption correction) [30]. The structures were solved using direct or Patterson methods in the program SHELXS-97 [31] and were refined by the full matrix least-squares method on *F2* with SHELXL-97. All the non-hydrogen atoms were refined anisotropically and all the C-bound hydrogen atoms were placed at calculated positions and refined isotropically. O-bound hydrogen atoms were located in difference Fourier maps and refined with distance restraint of O-H 0.84(2) Å. Drawings of the molecules were produced with XSEED [32]. Crystal data and refinement are summarized in Table 2.

### 3.9. Anti-AChE Assay

The anti-cholinesterase activities of the compounds were evaluated by Ellmann’s method with slight modifications, using acetylthiocholine as a substrate [33] and 5,5'-dithiobis[2-nitrobenzoic acid](DTNB). Sodium phosphate buffer (pH 8.0, 110 μL) was added into the 96 wells followed by sample solution (20 μL), DTNB (0.126 mM, 50 μL) and AChE enzyme (0.6 U/mL, 20 μL). The mixture was incubated for 50 minutes at 37 °C. The reaction was then initiated by the addition of acetylthiocholine iodide (0.120 mM, 50 μL). The hydrolysis of acetylthiocholine was monitored by the formation of yellow 5-thio-2-nitrobenzoate anion as the result of the reaction of DTNB with thiocholine, released by the enzymatic hydrolysis of acetylthiocholine, at a wavelength of 412 nm every 30 s for 25 minutes using a 96-well microplate plate reader (TECAN Infinite M200, Mannedorf, Switzerland). Test compounds were dissolved in analytical grade DMSO. Tacrine and propidium iodide were used as reference standards [34]. The reactions were performed in triplicate and monitored with a spectrophotometer. The percent inhibition of the enzyme activity due to the presence of increasing test compound concentration was obtained from the expression; 100 − (*vi/vo* × *100*), where *vi* is the initial rate calculated in the presence of inhibitors and *vo* is the enzyme activity.

### 3.10. Molecular Modeling Evaluations

The coordinates for the enzyme were those deposited in the Protein Data Bank for the human acetylcholinesterase (1B41) after eliminating the inhibitor (Fasciculin-2) and water molecules. The missing residues were built and polar hydrogen atoms were added using Discovery Studio 3.0 (Accelrys, Inc., San Diego, CA, USA). By default, solvation parameters and Kollman charges were assigned to all atoms of the enzyme using AutoDock Tools v.1.4. The 3D structures of the compounds were optimized according to the standard protocol in Discovery Studio 3.0. For docking studies, the latest version of AutoDock v.4.0 [35] was chosen because its algorithm allows full flexibility of small compounds. It has been shown to successfully reproduce many crystal structure complexes and includes an empirical binding free energy evaluation. Docking of compounds to AChE was carried out using the hybrid Lamarckian Genetic Algorithm. A grid box with the size of 178 Å × 200 Å × 194 Å and grid spacing of 0.375 Å was built to span the entire protein structure, in vacuo. The maximum number of energy evaluations was set to 25,000,000. Blind docking was used to predict structural features of compound binding. Resulting docked orientations within a root-mean square deviation of 1.5 Å were clustered. The lowest energy cluster reported by AutoDock for each compound was used for further analysis. All other parameters were maintained at their default settings. The structures of the complexes obtained were visualized and analyzed using Discovery Studio 3.0 and Ligplot 1.0 [36] to identify some specific interactions between the atoms of the compounds and the enzyme.

### 3.11. Antioxidant Activity

#### 3.11.1. DPPH (1,1-Diphenyl-2-picrylhydrazyl) Assay

The scavenging activities of the compounds on DPPH were measured according to a reported procedure [37]. The compounds showed final concentrations within the range of 0–25 μg/mL in methanol. One milliliter of 0.3 mM DPPH ethanol solution was added to sample solution (2.5 mL) of different concentrations and used as stock solutions for the test; meanwhile methanol (1 mL) was added to samples (2.5 mL) to make the blank solutions. The negative control (blank) consisted of DPPH solution (1 mL) plus methanol (2.5 mL). These solutions were allowed to react at room temperature for 30 min in the dark. The absorbance was read at 518 nm and converted into percentage antioxidant activity according to the following equation: % Inhibition = [(AB − AA)/AB] × 100. Where: AB: absorption of blank sample, AA: absorption of tested samples. The kinetics of DPPH scavenging activity was determined and the IC50 calculated using ascorbic acid as a positive control.

#### 3.11.2. FRAP Assay

The FRAP assay of the compounds performed using modified method as described by Benzie and Strain [38]. The stock solutions contained 300 mM acetate buffer (3.1 g CH_3_COONa·3H_2_O and 16 mL CH_3_COOH), pH 3.6, 10 mM TPTZ (2,4,6-tripyridyl-*s*-triazine) solution in 40 mM hydrochloric acid and 20 mM ferric chloride hexahydrate solution. The fresh working solution was prepared by mixing acetate buffer (25 mL), TPTZ (2.5 mL), and ferric chloride hexahydrate solution (2.5 mL). The temperature of the solution was raised to 37 °C before use and allowed to react with the FRAP solution (300 μL) in the dark. The colored product (ferrous tripyridyltriazine complex) was monitored at a wavelength of 593 nm. The standard curve was linear between 100 and 1,000 μM ferrous sulphate. Results are expressed in μM ferrous/g dry mass and compared with that of ascorbic acid and butylated hydroxytoluene.

### 3.12. Statistical Analysis

All values were reported as mean ± S.E.M. The statistical significance of differences between groups was assessed using one-way ANOVA. A value of *p* < 0.05 was considered significant.

## 4. Conclusions

Synthesized novel Schiff bases were observed to be potentially useful for acetyl-cholinesterase inhibition and possible treatment for AD. The compounds also showed strong free radical inhibitory activities. *In silico* molecular modeling revealed that the compounds may position themselves in the enzyme’s active-site gorge, interacting with residues in the peripheral anionic subsite (PAS) and acyl binding pocket (ABP).

## Supplementary Data

CCDC 857032 contains the supplementary crystallographic data for complex-4. These data can be obtained free of charge via http://www.ccdc.cam.ac.uk/conts/retrieving.html, or from the Cambridge Crystallographic Data Centre, 12 Union Road, Cambridge CB2 1EZ, UK; Fax: (+44) 1223-336-033; or Email: deposit@ccdc.cam.ac.uk. Detailed information can be accessed at: http://www.mdpi.com/1420-3049/17/3/2408/s1.

## References

[1] da Silva C.M., da Silva D.L., Modolo L.V., Alves R.B., de Resende M.A., Martins C.V.B., de Fatima A. (2011). Schiff bases: A short review of their antimicrobial activities. J. Adv. Res..

[2] Creaven B.S., Duff B., Egan D.A., Kavanagh K., Rosair G., Thangella V.R. (2010). Anticancer and antifungal activity of copper(II) complexes of quinolin-2(1H)-one-derived Schiff bases. Inorg. Chim. Acta.

[3] Ceyhan G., Çelik C., Uruş S., Demirtaş İ., Elmastaş M., Tümer M.  (2011). Antioxidant, electrochemical, thermal, antimicrobial and alkane oxidation properties of tridentate Schiff base ligands and their metal complexes. Spectrochim. Acta A Mol. Biomol. Spectrosc..

[4] Qiao X., Ma Z.-Y., Xie C.-Z., Xue F., Zhang Y.-W., Xu J.-Y. (2011). Study on potential antitumor mechanism of a novel Schiff Base copper(II) complex: Synthesis, crystal structure, DNA binding, cytotoxicity and apoptosis induction activiy. J. Inorg. Biochem..

[5] Xu D., Ma S., Du G., He Q., Sun D. (2008). Synthesis, characterization, and anticancer properties of rare earth complexes with Schiff base and o-phenanthroline. J. Rare Earth..

[6] Rice-Evans C. Implications of the mechanisms of action of tea polyphenols as antioxidants *in vitro* for chemoprevention in humans. Proceedings of the Society for Experimental Biology and Medicine.

[7] Clemetso C.A.B., Andersen L., Clemetso C.A.B., Andersen L. (1966). Plant Polyphenols as Antioxidants for Ascorbic Acid.

[8] Sun-Waterhouse D., Chen J., Chuah C., Wibisono R., Melton L.D., Laing W. (2009). Kiwifruit-based polyphenols and related antioxidants for functional foods: Kiwifruit extract-enhanced gluten-free bread. Int. J. Food Sci. Nutr..

[9] Kuhn D.J., Lam W.H., Kazi A., Daniel K.G., Song S.J., Chow L.M.C. (2005). Synthetic peracetate tea polyphenols as potent proteasome inhibitors and apoptosis inducers in human cancer cells. Front Biosci..

[10] Samoylenko O., Zaletok S., Orlovsky O., Gogol S., Klenov O., Shapochka D. (2011). Additive antitumor effect of plant polyphenols and a synthetic inhibitors of polyamines biosynthesis. Breast.

[11] Claudine M., Andrzej M., Augustin S. (2005). Polyphenols and prevention of cardiovascular diseases. Curr. Opin. Lipidol..

[12] Capasso R., Evidente A., Tremblay E., Sala A., Santoro C., Cristinzio G. (1994). Direct and mediated effects on *Bactrocera oleae* (Gmelin) (Diptera, Tephritidae) of natural polyphenols and some of related synthetic compounds: Structure-activity relationships. J. Chem. Ecol..

[13] Luchsinger J.A., Mayeux R. (2004). Dietary factors and Alzheimer’s disease. Lancet Neurol..

[14] Dai Q., Borenstein A.R., Wu Y., Jackson J.C., Larson E.B. (2006). Fruit and vegetable juices and Alzheimer’s disease: The *Kame* Project. Am. J. Med..

[15] Bourn D., Prescott J. (2002). A comparison of the nutritional value, sensory qualities, and food safety of organically and conventionally produced foods. Crit. Rev. Food Sci. Nutr..

[16] Arts I.C., Hollman P.C. (2005). Polyphenols and disease risk in epidemiologic studies. Am. J. Clin. Nutr..

[17] El-Ansary A.L., Abdel-Fattah H.M., Abdel-Kader N.S. (2011). Synthesis, spectral, thermal and magnetic studies of Mn(II), Ni(II) and Cu(II) complexes with some benzopyran-4-one Schiff bases. Spectrochim. Acta A Mol. Biomol. Spectrosc..

[18] Khan T.A., Naseem S., Khan S.N., Khan A.U., Shakir M. (2009). Synthesis and spectral characterization of 14- and 16-membered tetraazamacrocyclic Schiff base ligands and their transition metal complexes and a comparative study of interaction of calf thymus DNA with copper(II) complexes. Spectrochim. Acta A Mol. Biomol. Spectrosc..

[19] Nath M., Saini P.K., Kumar A. (2010). New di- and triorganotin(IV) complexes of tripodal Schiff base ligand containing three imidazole arms: Synthesis, structural characterization, anti-inflammatory activity and thermal studies. J. Organomet. Chem..

[20] Issa R.M., Khedr A.M., Rizk H.F. (2005). UV-vis, IR and 1H NMR spectroscopic studies of some Schiff bases derivatives of 4-aminoantipyrine. Spectrochim. Acta A Mol. Biomol. Spectrosc..

[21] Pang S., Liang Y. (2001). Studies on charge transfer properties from mixture of Schiff base and zinc complex in Langmuir-Blodgett film by UV-vis absorption and Fourier transform infrared spectroscopy. Spectrochim. Acta A Mol. Biomol. Spectrosc..

[22] Suleiman Gwaram N., Khaledi H., Mohd Ali H., Robinson W.T., Abdulla M.A. (2010). *N'*-[1-(5-Bromo-2-hydroxyphenyl)ethylidene]-3,4,5-trihydroxybenzohydrazide dimethyl sulfoxide solvate trihydrate. Acta Crystallogr..

[23] Heider E.M., Harper J.K., Grant D.M., Hoffman A., Dugan F., Tomere D.P., O’Neille K.L. (2006). Unusual antioxidant activity in a benzoic acid derivative: A proposed mechanism for citrinin. Tetrahedron.

[24] Kadoma Y., Atsumi T., Okada N., Ishihara M., Yokoe I., Fujisawa S. (2007). Radical-scavenging activity of the reaction products of isoeugenol with thiol, thiophenol, mercaptothiazoline or mercaptomethylimidazole using the induction period method. Molecules.

[25] Kryger G., Harel M., Giles K., Toker L., Velan B., Lazar A., Kronman C., Barak D., Ariel N., Shafferman A. (2000). Structures of recombinant native and e202q mutant human acetylcholinesterase complexed with the snake-venom toxin fasciculin-ii. Acta Crystallogr. D Biol. Crystallogr..

[26] Wiesner J., Kriz Z., Kuca K., Jun D., Koca J. (2007). Acetylcholinesterases—The structural similarities and differences. J. Enzyme Inhib. Med. Chem..

[27] Heo B.G., Park Y.S., Chon S.U., Lee S.Y., Cho J.Y., Gorinstein S. (2007). Antioxidant activity and cytotoxicity of methanol extracts from aerial parts of Korean salad plants. Biofactors.

[28] Khaledi H., Alhadi A.A., Yehye W.A., Ali H.M., Abdulla M.A., Hassandarvish P. (2011). Antioxidant, cytotoxic activities, and structure-activity relationship of gallic acid-based indole derivatives. Arch. Pharm..

[29] Stockdale M., Selwyn M.J. (1971). Effects of ring substituents on the activity of phenols as inhibitors and uncouplers of mitochondrial respiration. Eur. J. Biochem..

[30] (2007). Bruker APEX2 and SAINT.

[31] Sheldrick G.M. (2008). A short history of SHELX. Acta Crystallogr. A.

[32] Barbour L.J. (2001). X-Seed—A software tool for supramolecular crystallography. J. Supramol. Chem..

[33] Guilhermino L., Lopes M.C., Carvalho A.P., Soares A.M.V.M. (1996). Inhibition of acetylcholinesterase activity as effect criterion in acute test with juvenile *Daphnia magna*. Chemosphere.

[34] Laskwoski R.A. (2001). PDBsum: Summaries and analyses of PDB structure. Nucleic Acids Res..

[35] Morris G.M., Goodsell D.S., Halliday R.S., Huey R., Hart W.E., Belew R.K., Olson A.J. (1998). Automated docking using a Lamarckian genetic algorithm and empirical binding free energy function. J. Comput. Chem..

[36] Wallace C.A., Laskowski A.R., Thornton M.J. (1995). LIGPLOT: A program to generate schematic diagrams of protein-ligand interactions. Protein Eng..

[37] Choi W.C., Kim S.C., Hwang S.S., Choi B.K., Ahn H.J., Lee M.Y., Park S.H., Kim S.K. (2002). Antioxidant activity and free radical scavenging capacity between Korean medicinal plants and flavonoids by assay-guided comparison. Plant Sci..

[38] Benzie I.F.F., Strain J.J. (1999). Ferric reducing/antioxidant power assay: Direct measure of total antioxidant activity of biological fluids and modified version for simultaneous measurement of total antioxidant power and ascorbic acid concentration. Method Enzymol..

