# Supplementary material for: Synthesis, Characterization, X-ray Crystallography, Acetyl Cholinesterase Inhibition and Antioxidant Activities of Some Novel Ketone Derivatives of Gallic Hydrazide-Derived Schiff Bases
Source: Molecules. 2012 Feb 28;17(3):2408–27. doi: 10.3390/molecules17032408 (PMC6268099; doi:10.3390/molecules17032408)

checkCIF/PLATON report

You have not supplied any structure factors. As a result the full set of tests cannot be run.

No syntax errors found. CIF dictionary Interpreting this report

Datablock: sn1lig_552_0m

```
Bond precision:  C-C = 0.0046 A          Wavelength=0.71073

Cell:            a=7.769(9)    b=15.509(19)       c=13.162(16)
                 alpha=90      beta=103.85(2)     gamma=90
Temperature:     296 K
```

	Calculated	Reported
Volume	1540(3)	1540(3)
Space group	P 21/n	P2(1)/n
Hall group	-P 2yn	?
Moiety formula	C16 H16 N2 O6, H2 O	?
Sum formula	C16 H18 N2 O7	C16 H18 N2 O7
Mr	350.32	350.32
Dx,g cm-3	1.511	1.511
Z	4	4
Mu (mm-1)	0.120	0.120
F000	736.0	736.0
F000’	736.44	
h,k,lmax	9,18,15	9,18,15
Nref	2869	2851
Tmin,Tmax	0.993,0.999	0.967,0.998
Tmin’	0.967	

```
Correction method= MULTI-SCAN

Data completeness= 0.994          Theta(max)= 25.500

R(reflections)= 0.0546( 1612)     wR2(reflections)= 0.1345( 2851)

S = 1.003                  Npar= 247
```

The following ALERTS were generated. Each ALERT has the format
test-name_ALERT_alert-type_alert-level.
Click on the hyperlinks for more details of the test.

Alert level C

```
PLAT148_ALERT_3_C su on the      a  - Axis is (Too) Large ........      0.009 Ang.
PLAT148_ALERT_3_C su on the      b  - Axis is (Too) Large ........     0.0190 Ang.
PLAT148_ALERT_3_C su on the      c  - Axis is (Too) Large ........      0.016 Ang.
PLAT340_ALERT_3_C Low Bond Precision on  C-C Bonds ...............     0.0046 Ang
```

Alert level G

```
PLAT002_ALERT_2_G Number of Distance or Angle Restraints on AtSite         11
PLAT005_ALERT_5_G No _iucr_refine_instructions_details in CIF ....          ?
PLAT128_ALERT_4_G Alternate Setting of Space-group P21/c   .......      P21/n
PLAT860_ALERT_3_G Note: Number of Least-Squares Restraints .......          6
```

```
   0 ALERT level A = Most likely a serious problem - resolve or explain
   0 ALERT level B = A potentially serious problem, consider carefully
   4 ALERT level C = Check. Ensure it is not caused by an omission or oversight
   4 ALERT level G = General information/check it is not something unexpected

   0 ALERT type 1 CIF construction/syntax error, inconsistent or missing data
   1 ALERT type 2 Indicator that the structure model may be wrong or deficient
   5 ALERT type 3 Indicator that the structure quality may be low
   1 ALERT type 4 Improvement, methodology, query or suggestion
   1 ALERT type 5 Informative message, check
```

It is advisable to attempt to resolve as many as possible of the alerts in all categories. Often the minor alerts point to easily fixed oversights, errors and omissions in your CIF or refinement strategy, so attention to these fine details can be worthwhile. In order to resolve some of the more serious problems it may be necessary to carry out additional measurements or structure refinements. However, the purpose of your study may justify the reported deviations and the more serious of these should normally be commented upon in the discussion or experimental section of a paper or in the "special_details" fields of the CIF. checkCIF was carefully designed to identify outliers and unusual parameters, but every test has its limitations and alerts that are not important in a particular case may appear. Conversely, the absence of alerts does not guarantee there are no aspects of the results needing attention. It is up to the individual to critically assess their own results and, if necessary, seek expert advice.

Publication of your CIF in IUCr journals

A basic structural check has been run on your CIF. These basic checks will be run on all CIFs submitted for publication in IUCr journals (*Acta Crystallographica*, *Journal of Applied Crystallography*, *Journal of Synchrotron Radiation*); however, if you intend to submit to *Acta Crystallographica Section C* or *E*, you should make sure that full publication checks are run on the final version of your CIF prior to submission.

Publication of your CIF in other journals

Please refer to the *Notes for Authors* of the relevant journal for any special instructions relating to CIF submission.

PLATON version of 18/07/2011; check.def file version of 04/07/2011

Datablock sn1lig_552_0m - ellipsoid plot

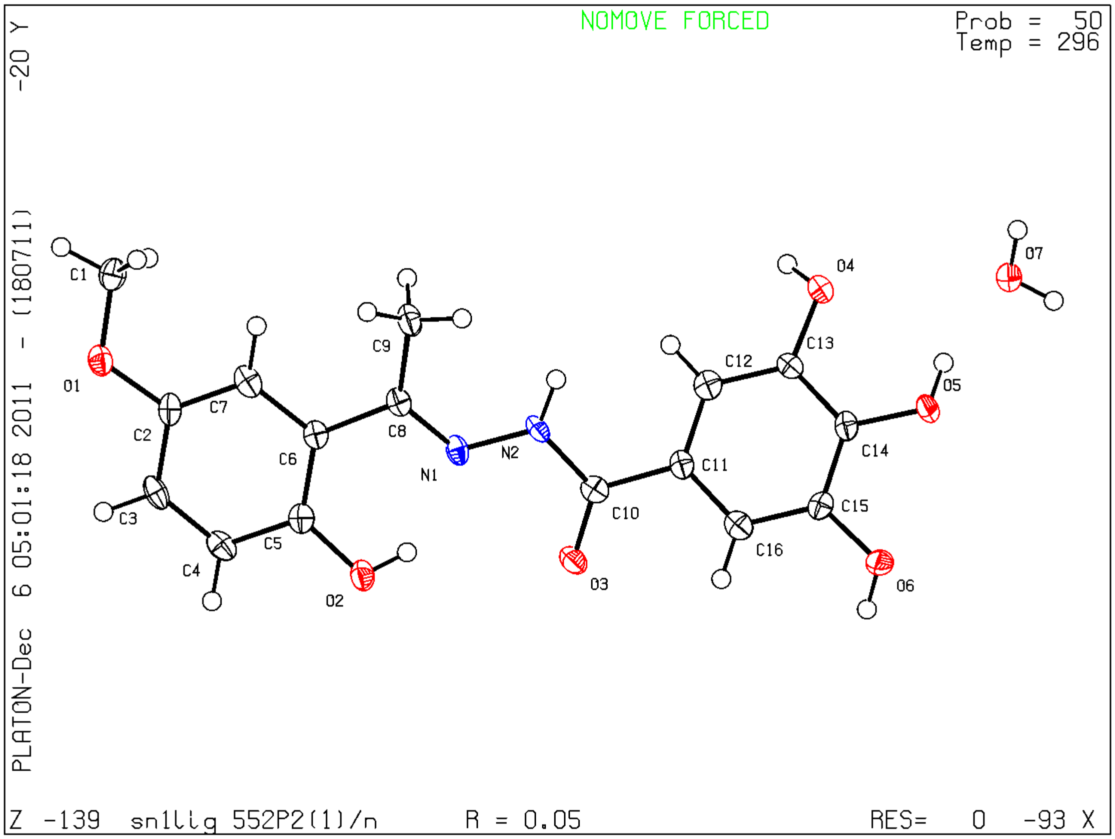

Supplement: Supplementary file 1 [file molecules-17-02408-s001.pdf]
